# The DNA Glycosylase NEIL2 Suppresses *Fusobacterium*-Infection-Induced Inflammation and DNA Damage in Colonic Epithelial Cells

**DOI:** 10.3390/cells9091980

**Published:** 2020-08-28

**Authors:** Ibrahim M. Sayed, Anirban Chakraborty, Amer Ali Abd El-Hafeez, Aditi Sharma, Ayse Z. Sahan, Wendy Jia Men Huang, Debashis Sahoo, Pradipta Ghosh, Tapas K. Hazra, Soumita Das

**Affiliations:** 1 Department of Pathology, University of California, San Diego, CA 92093, USA; i4ibrahim@health.ucsd.edu (I.M.S.); a5sharma@health.ucsd.edu (A.S.); asahan@health.ucsd.edu (A.Z.S.); 2 Department of Medical Microbiology and Immunology, Faculty of Medicine, Assiut University, Assiut 71515, Egypt; 3 Department of Internal Medicine, University of Texas Medical Branch, Galveston, TX-77555, USA; anibiotech1111@gmail.com (A.C.); tkhazra@utmb.edu (T.K.H.); 4 Department of Cellular and Molecular Medicine, University of California, San Diego, CA 92093, USA.; aam002@health.ucsd.edu (A.A.A.E.-H.); wendyjmhuang@health.ucsd.edu (W.J.M.H.); prghosh@health.ucsd.edu (P.G.); 5 Department of Pediatrics, University of California, San Diego, CA 92093, USA; dsahoo@health.ucsd.edu; 6 Department of Computer Science and Engineering, Jacob’s School of Engineering, La Jolla, CA 92093, USA; 7 Department of Medicine, University of California, San Diego, CA 92093, USA; 8 Moores Cancer Center, University of California, San Diego, CA 92093, USA

**Keywords:** *Fusobacterium nucleatum*, inflammation, enteroid, enteroid-derived monolayer, DNA damage, base-excision repair, colorectal cancer, NEIL2, genomic instability, cancer development

## Abstract

Colorectal cancer (CRC) is the third most prevalent cancer, while the majority (80–85%) of CRCs are sporadic and are microsatellite stable (MSS), and approximately 15–20% of them display microsatellite instability (MSI). Infection and chronic inflammation are known to induce DNA damage in host tissues and can lead to oncogenic transformation of cells, but the role of DNA repair proteins in microbe-associated CRCs remains unknown. Using CRC-associated microbes such as *Fusobacterium nucleatum* (*Fn*) in a coculture with murine and human enteroid-derived monolayers (EDMs), here, we show that, among all the key DNA repair proteins, NEIL2, an oxidized base-specific DNA glycosylase, is significantly downregulated after *Fn* infection. *Fn* infection of NEIL2-null mouse-derived EDMs showed a significantly higher level of DNA damage, including double-strand breaks and inflammatory cytokines. Several CRC-associated microbes, but not the commensal bacteria, induced the accumulation of DNA damage in EDMs derived from a murine CRC model, and *Fn* had the most pronounced effect. An analysis of publicly available transcriptomic datasets showed that the downregulation of NEIL2 is often encountered in MSS compared to MSI CRCs. We conclude that the CRC-associated microbe *Fn* induced the downregulation of NEIL2 and consequent accumulation of DNA damage and played critical roles in the progression of CRCs.

## 1. Introduction

Colorectal cancer (CRC) is the third-most prevalent cancer and the fourth-most frequent cause of cancer death in the USA and around the world, according to GLOBOCAN 2018 data and the American Cancer Society, 2017 [1]. CRC is a multifactorial disease that is affected by genetic, epigenetic, and environmental factors, such as dietary lifestyle, physical activity, obesity, smoking, alcohol consumption, and alteration of gut microbiota [2,3,4,5,6,7]. Epigenetic alterations in CRC include aberrant DNA methylation, chromatin modifications, and noncoding microRNA expression [8]. For the genetic subtype of CRC, the presence or absence of mutations in the DNA mismatch repair system is well-documented [9]. However, this hypermutable subtype or deficient mismatch repair (dMMR) system is responsible for only 15% of CRCs, which is known as microsatellite instable (MSI) CRC [10]. The defect in the MMR pathway leads to MSI, and it is a common etiologic factor for the development of CRC, such as hereditary non-polyposis colon cancer (HNPCC), also known as Lynch syndrome [11]. The remaining 85% of the CRCs do not exhibit mutations in the mismatch repair system, and these CRCs are known as proficient mismatch repair (pMMR) or microsatellite stable (MSS) CRCs [12,13]. 

The persistent microbial infection triggers the induction of chronic inflammation and DNA damage and these two cellular processes, in combination, are the major risk factors for cancer development, including CRC. The alteration of gut microbes can exert a profound influence on host physiology by inducing a chronic inflammatory state, which affects stem cell precursors and the production of toxic metabolites leading to the development of CRC [14,15]. Numerous bacterial species, such as *Bacteroides fragilis*, *Helicobacter pylori*, *Escherichia coli*, *Enterococcus faecalis*, *Clostridium septicum* and *Fusobacterium nucleatum* play roles in the pathogenesis of CRC [16,17]. *Fusobacterium nucleatum (Fn)* is a pathogenic anaerobic gram-negative gut microbe that is also associated with CRC cases [18,19]. *Fn* is associated with more than 50% of CRCs, including MSI and MSS cancers, with a higher prevalence in the proximal colon than distal colon [20,21,22]. Moreover, high levels of *Fn* in CRC patients’ tissues was associated with poor patient outcome and recurrence of infection in post-chemotherapy [23]. Several possible mechanisms have been proposed, such as binding of the FadA adhesin of *Fn* to E-cadherin leading to activation of Wnt/β-catenin signaling, which then causes overexpression of Wnt genes, oncogenes c-Myc, Cyclin D1, and inflammatory genes involved in CRC progression [19,24]. *Fn* selectively promotes the growth of cancerous cells by inducing Wnt/β-catenin modulator Annexin A1 [25]. A recent study showed that *Fn* caused DNA damage and promoted cell proliferation by the Ku70/p53 pathway in oral cancer cells [26]. 

Pathogenic bacteria induce the release of reactive oxygen and nitrogen species (RONS), which eventually can lead to mutagenic oxidized DNA base lesions [27]. Host cells respond to DNA damage by initiating DNA damage response (DDR) following infection. Failure of DDR can lead to the accumulation of mutations, inducing genome instability, and oncogene activation resulting in cancer [28,29]. However, to date, there is no report on how colon cancer-associated bacteria such as *Fn* infection can impact DNA damage and inflammation and how the BER proteins play a critical role in the etiology of colorectal cancer. ROS-induced oxidative DNA base lesions are predominantly repaired via the highly conserved base excision repair (BER) pathway, which is initiated by DNA glycosylases. [30]. Among the five oxidized base-specific DNA glycosylases, OGG1 and NTH1 remove oxidized purines and pyrimidines, respectively, from duplex DNA. *E. coli* Nei-like (NEIL1-3) proteins are a distinct family of DNA glycosylases that remove both oxidized purines and pyrimidines. They initiate the excision of lesions present in a single-stranded region, a replication fork, or transcription bubble mimic [31,32,33,34,35,36]. The reduced expression of NEIL1, NEIL2, and elevated expression of NEIL3 is involved in the progression of several types of cancer as a consequence of somatic mutation load [37]. The decreased level or loss of activity of NEIL2 and, to a lesser extent, NEIL1, is a risk factor for the progression of cancers such as lung cancer and squamous cell carcinoma in the oral cavity [38,39,40,41], and poor survival in patients with estrogen-receptor-positive breast cancer [42]. BER is the major pathway for the repair of such DNA lesions, and thereby a robust DNA damage response through BER can reduce the risk of colon carcinogenesis [43]. Recently, we have shown that *Helicobacter pylori* infection leads to degradation of NEIL2 and subsequent accumulation of oxidative DNA damage, a plausible cause of gastric cancer [44]. This result is in agreement with our earlier studies showing that the *Neil2*-null mice are highly sensitive to inflammatory stress and also accumulate oxidized DNA bases in their genome [31]. 

Here, we determined the effect of *Fn* infection on the host DNA repair pathways and analyzed the expression level of vital proteins using stem cell-based enteroid-derived monolayers (EDMs). Among the DNA repair proteins, MSH2/MSH6 (involved in the MMR pathway) and Ku 70 (involved in nonhomologous end joining (NHEJ)) were upregulated, and only NEIL2 (BER) was downregulated following infection. *Fn* infection increased the inflammatory cytokines and induced DNA damage in colonic EDMs. Consequently, *Fn* infection leads to double-strand DNA breaks, which were more prominent when the BER protein (NEIL2) was depleted. This study demonstrated that *Fn* infection exerted a differential effect on DNA repair pathways in colonic EDMs. It further provided novel insight into the critical role of NEIL2 in controlling DNA damage accumulation and inflammation following *Fn* infection and, thus, established a plausible link between impaired DNA repair, uncontrolled inflammation, and CRC progression. 

## 2. Materials and Methods

All experiments involving human and animal subjects were performed following the relevant guidelines and regulations of the University of California, San Diego, CA, USA and the National Institutes of Health (NIH).

### 2.1. Animals

The *Neil2* knockout (KO) C57BL/6 mice were generated and characterized as previously described [32]. The breeding and maintenance of these mice were carried out as per the approved guidelines of the Animal Care and Ethics Committee of UTMB, Galveston, TX, USA (Hazra protocol 0606029D). APC^Min/+^ (Adenomatous polyposis coli) mice were bred, maintained, and subsequently used for multiple experiments following the University of California San Diego Institutional Animal Care and Use Committee (IACUC) policies (Protocol #S18086). 

### 2.2. Bacterial Cultures

*Fusobacterium nucleatum (Fn)* (ATCC-25586) was obtained from ATCC. *Fn* was cultured anaerobically at 37 °C for 48 h in the chopped cooked meat media (Anaerobic Systems, Morgan Hill, CA, USA) inside an anaerobic chamber containing an anaerobic gas kit generating system (MGC, AnaeroPACK System, New York, NY, USA). Colon enteroid-derived monolayer (EDM) was infected with *(Fn)* at a multiplicity of infection (moi) of 100 for 24 h. *Escherichia coli K12 strain DH10B* (ATCC-PTA5105) was cultured on Luria Broth (LB) agar and LB broth and used to infect EDM at a moi of 100 for 24 h. Adherent-invasive *Escherichia coli* strain LF82 (AIEC-LF82), isolated from the specimens of Crohn’s disease patients, was obtained from Arlette Darfeuille-Michaud (Clermont-Ferrand, France) and was cultured as previously described [45]. EDM was infected with LF82 at a moi of 100 for 24 h. *H. pylori* strain (ATCC-26695) was incubated with the Brucella broth supplemented with 10% fetal bovine serum (FBS) in a shaker at 37 °C in 10% CO_2_. Colon EDMs were infected with *H. pylori* at moi 100 for 24 h.

### 2.3. Isolation and Maintenance of Colonic Organoids 

Colon organoids were isolated from the colonic tissue specimens of wild-type (WT) C57BL/6 mice, *Neil2* KO mice, *CPC-APC*^Min/+^ mice, healthy human subjects, and colorectal cancer (CRC) patients, as described previously [45,46,47,48,49]. Briefly, colon tissues were digested by collagenase type I (2 mg/mL; Life Technologies Corporation, Grand Island NY, USA) containing gentamicin (50 μg/mL, Life Technologies Corporation). The digested tissues were incubated at 37 °C for 30–40 min cycles with intervals of 10 min each. The tissue specimens were subjected to vigorous pipetting between successive incubations and were constantly monitored to confirm the dislodgement of the intestinal crypts from the tissues. After the separation of 80% of the colon crypts, the collagenase was inactivated with media (DMEM/F12 with HEPES, 10% fetal bovine serum (FBS) (Sigma-Aldrich, MO, USA), and the colon crypts were filtered using a 70-μm cell strainer. The number of live cells was measured in a hemocytometer following staining by trypan blue (Sigma-Aldrich). The cells were suspended in the basement membrane matrix (matrigel) (Corning Inc., Kennebunk, ME, USA) supplemented with 50% stem cell-enriched conditioned medium (CM) with WNT 3a, R-spondin, and Noggin. Y27632 (ROCK inhibitor, 10 μM) and SB431542 (an inhibitor for TGF-β type I receptor, 10 μM) were added to the media to support the growth of organoids. For the human enteroids, media and supplements were obtained from the HUMANOID CoRE (UC San Diego, CA, USA), as reported previously [45]. 

### 2.4. The Preparation of Enteroid-Derived Monolayers (EDMs) 

EDMs were prepared from colonic enteroids as previously described [45,50]. Briefly, the enteroids were trypsinized, and the isolated cells were filtered, counted, resuspended in 5% CM, and 2 × 10^5^ cells (per well) were plated in 0.4-μm polyester membrane Transwell (Corning, Cat #3470, MO, USA) precoated with matrigel (1:40 dilution). The cells were then allowed to differentiate for 48-72 h. Transepithelial electrical resistance (TEER) and Lgr5 were used as markers for the quality and differentiation of EDMs [45,47]. Differentiated EDMs were challenged with different microbes, as described earlier. The supernatant was collected from the basolateral part of the Transwell for cytokine analysis, and the cells were lysed for RNA extraction to check for the expression of target genes by qPCR.

For murine colonic organoids, we used 3 age and gender-matched mice in each group, and for human colonic organoids, we used biopsy specimens from 3 adult healthy human subjects. Each organoid line isolated from one mouse or from one human subject was biobanked and differentiated to EDM. Therefore, each EDM was derived from one mouse, and there were three different cultures from WT mice and three different cultures from NEIL2 KO mice used in this study. Additionally, there were 3 different human organoid and EDM cultures used in this study. 

### 2.5. RNA Isolation and qPCR for Gene Expression Analysis

RNA was extracted from uninfected EDMs, and EDMs infected with *Fn*, *E. coli* K12-, and LF82 using Quick-RNA MicroPrep Kit (Zymo Research, Irvine, CA, USA) according to the manufacturer’s instructions. cDNA was synthesized using the qScript™ cDNA SuperMix (Quantabio, Beverly, MA, USA). Quantitative PCR (qPCR) was carried out using 2× SYBR Green qPCR Master Mix (Biomake, Houston, TX, USA) for target genes, and the data was normalized to the housekeeping 18s rRNA gene using the ΔΔCt method. Primers were designed using the National Center for Biotechnology Information (NCBI) Primer Blast software (https://www.ncbi.nlm.nih.gov/tools/primer-blast/index.cgi) and the Roche Universal Probe Library Assay Design software https://lifescience.roche.com/en_us/brands/universal-probe-library.html#assay-design-center (Table 1).

### 2.6. Proteome Profiler Array

To assess the cytokine levels following *Fn* infection, a proteome profiler array (human cytokine array; R & D Systems, Minneapolis, MN, USA) was used according to the manufacturer’s instructions. Briefly, the capture antibody of cytokines and chemokines were spotted on nitrocellulose membranes precoated with 40 cytokines. The samples/secondary antibody mixture was added to the immobilized capture antibodies on the membrane following the manufacturer’s protocols. After washing, the streptavidin-horseradish peroxidase and chemiluminescent reagents were added to the membrane. The detected signal was proportional to the amount of cytokine bound. Quantification of specific cytokines was performed using ELISA.

### 2.7. The Measurement of Interlekin (IL)-8 Cytokine by ELISA

Supernatants were collected from WT, *NEIL2* KO EDMs, and APC^Min/+^ polyp either uninfected or infected with *Fn*. IL-8 (KC) was measured using the mouse CXCL1/KC DuoSet ELISA kit (R & D Systems) according to the manufacturer’s instructions. The level of IL-8 was compared between *Fn*-infected EDMs vs. the uninfected EDMs from WT, *Neil2* KO mice, and APC^Min/+^ polyp.

### 2.8. Immunoblot for Detection of Base Excision Repair and Cellular Signaling Proteins

Whole-cell lysates were prepared using the RIPA buffer (50-mM Tris (pH 7.4), 150-mM NaCl, 1% NP-40, 0.25% Na-deoxycholate, and 1-mM EDTA with protease inhibitor cocktail (Sigma, MO, USA) from uninfected and *Fn*-infected WT and *NEIL2* KO EDMs and subsequently tested for the expression levels of BER proteins. The proteins in the whole-cell extracts from EDMs were separated onto a Bio-Rad 4–20% gradient Bis-Tris Gel, then electrotransferred on nitrocellulose (0.45-μm pore size; GE Healthcare, Chicago, IL, USA) membrane using 1X Bio-Rad transfer buffer. The membranes were blocked with 5% *w/v* skimmed milk in TBST buffer (1X Tris-buffered Saline and 0.1% Tween 20), then immunoblotted with appropriate antibodies (NEIL2, NTH1, and OGG1; all anti-rabbit; and in-house antibodies used in 1:500 dilutions). Glyceraldehyde 3-phosphate dehydrogenase (GAPDH) and histone deacetylase 2 (HDAC2) (GeneTex, Irvine, CA, USA) were used as loading controls. The membranes were extensively washed with 1% TBST, followed by incubation with anti-isotype secondary antibody (GE Healthcare) conjugated with horseradish peroxidase in 5% skimmed milk at room temperature. Subsequently, the membranes were further washed three times (10 min each) in 1% TBST and developed using ECL^TM^ Western Blotting Detection Reagents (RPN2209, GE Healthcare) and imaged immediately. For the detection of phosphorylated Ataxia telangiectasia-mutated kinase (pATM) and total ATM, cell lysates were separated on 8% SDS-PAGE and transferred to Polyvinylidine Fluride (PVDF) membranes (Millipore, Billerica, MA, USA). Membranes were blocked with phosphate buffered saline (PBS) supplemented with 5% nonfat milk or bovine serum albumin and then incubated sequentially with primary and secondary antibodies. The dilution of the primary antibodies was as follows: anti-p-ATM, 1:1000; anti-ATM, 1-1000; and anti-tubulin, 1–1000 (used as a loading control). All the Western blot images were quantified using ImageJ automated digitizing system based on three independent gel images (*n* = 3).

### 2.9. Assessment of γH2AX Phosphorylation by Immunofluorescence (IF)

Either uninfected or *Fn*-infected EDMs from WT and *Neil2* KO mice were fixed with 4% paraformaldehyde (PFA) at room temperature for 15 min, washed once with 1X phosphate-buffered saline, permeabilized, and blocked with IF buffer (0.1% Triton TX-100 and 2-mg/mL bovine serum albumin (BSA) in PBS) for 1 h. Samples were then incubated with anti-p-**γ**H2AX (Santa Cruz biotechnology, Inc. Santa Cruz, CA, USA 1:500). Incubation with the primary antibody was performed at 4 °C overnight. After three consecutive washes with PBS (10 min each), the EDMs were incubated with secondary antibodies at a dilution of 1:500 for 60 min at room temperature in the dark. Washes were performed as before, and the membranes were dried and mounted in gel/mount antifade aqueous mounting medium (Biotium, Fremont, CA, USA with coverslips. Images were acquired using a Leica CTR4000 Confocal Microscope with a 63X objective. Z-stack images were obtained by imaging approximately 4-μm-thick sections of cells in all channels. After capturing the image, the fluorescence intensity of γH2AX staining/cell was calculated using ImageJ. 

### 2.10. Analysis of Accumulation of DNA Strand Break by Long Amplicon Quantitative PCR (LAqPCR)

Genomic DNA (gDNA) was extracted from WT, *NEIL2-*KO, and APC^Min/+^ EDMs, either uninfected or infected with *Fn*, using a Gentra Puregene Cell Kit (Qiagen, Hilden, Germany) following the manufacturer’s instructions. Accumulation of DNA strand-break was assessed in both transcribed genes (*polß and globin*) following the protocol described previously [31,44]. Briefly, the extracted gDNA was quantified by Pico Green (Molecular Probes, ThermoFischer, Waltham, MA, USA). The gene-specific DNA strand-break accumulation was measured by long amplicon quantitative-PCR (LA-qPCR) by amplifying a 6.5-kb region of *polß* and the 8.7-kb region of globin genes, according to the following conditions: 94 °C for 30 s (94 °C for 30 s, 55–60 °C for 30 s depending on the oligo annealing temperature, and 65 °C for 10 min) for 25 cycles and 65 °C for 10 min. In principle, strand break (SB) following the excision of the oxidized DNA base by DNA glycosylases or DNA double-strand breaks, in general, will cause stalling of the Taq DNA polymerase, since they may not be able to bypass the strand break site. Thus, a decreased level of the long amplicon PCR product would reflect a higher level of DNA SBs. A small DNA fragment for each gene was also amplified to normalize the amplification of large fragments. Amplification of a smaller fragment should be similar for the samples because of a lower probability of SB formation in a shorter fragment. Thus, amplification of the short fragment is used for normalization of the differential amplification of the long fragment. The amplified products were visualized on agarose gels and quantified using the ImageJ automated digitizing system (NIH) based on at least two independent replicates. The extent of damage was calculated in terms of the relative band intensity with WT untreated subjects considered as 100. The oligo sequences used in the study are listed in Table 2.

### 2.11. Measurement of Oxidative DNA/RNA Damage

The level of damaged bases was quantified using the DNA/RNA Oxidative Damage ELISA Kit (Cayman Chemical, Ann Arbor, Michigan, USA, Clone 7E6.9) according to the manufacturer’s instructions and previous reports [51,52]. Briefly, this assay is an ELISA immunoassay that detects oxidized the guanine species 8-hydroxy-2′-deoxaguanosine from DNA and 8-hydroxyguanine from either DNA or RNA. Briefly, this assay is based on a competition between oxidatively damaged guanosine species and 8-hydroxy deoxaguanosine acetylcholinesterase conjugate (DNA/RNA oxidative damage tracer) for a limited amount of DNA/RNA Oxidative Damage Monoclonal antibody. The amount of tracer, which is held constant, that will bind to the monoclonal antibody is inversely proportional to the concentration of oxidatively damaged guanine species in the well. The secondary antibody (goat polyclonal anti-mouse IgG) has been previously attached to the well and will react with the antibody oxidatively damaged guanine complex. The reaction is developed by the addition of Ellman’s reagent that has substrated to acetylcholinesterase. The intensity of the color is determined spectrophotometrically at wavelengths 405–420 and is proportional to the amount of oxidative damage tracer bound to the well, which is inversely proportional to the amount of free 8-hydroxy guanosine present in the well. Standard curves were developed using standards provided in the kits, and the standards were diluted in the same culture media of the infection experiment to confirm that the matrix of the standards and the samples is the same. We used this assay to compare the level of oxidatively damaged bases in WT and *NEIL2* KO colon EDMs either uninfected or *Fn*-infected. Additionally, we used this technique to assess the level of DNA/RNA damage generated after infection with several bacteria (*E. coli* K12, *AIEC LF-82*, *H. pylori*, and *E. coli* NC101).

### 2.12. Lactate Dehydrogenase (LDH) Assay

Supernatants collected from WT and *NEIL2* KO colonic EDMs (either uninfected or *Fn*-infected) were assayed for LDH using a LDHGlo ^TM^ Cytotoxicity Assay kit (Promega, Madison, WI, USA) according to the manufacturer’s instructions, as described previously [45]. Briefly, supernatants were diluted in LDH storage buffer, and then, an equal volume of LDH detection reagent was added to the diluted sample. The LDH activity was determined by measurement of the degree of luminescence. LDH-positive control (purified lactate dehydrogenase from rabbit muscle) was included in each assay. Serial dilutions from the LDH-positive control (32–0.5 mU/mL) were done and included in this assay. Culture media was used to determine the medium background.

### 2.13. Analysis of Datasets with the CRC Patients

Publicly available CRC datasets GSE13067, GSE13294, GSE26682, and GSE18088 were downloaded from the National Center for Biotechnology Information (NCBI) Gene Expression Omnibus website (GEO) (PMID: 11752295, 15608262, and 23193258) and renormalized using RMA (robust multichip average) (PMID: 12582260, and 12925520). MSI/MSS status was manually annotated by going through GEO descriptions. Boxplots of NEIL2, NEIL1, OGG1, NTHL1, APEX1, POLB, and LIG3 expression values were computed using Hegemon (hierarchical exploration of gene expression microarrays online) analysis framework (PMID: 22308455, 22081019, and 26789870). Hegemon is a web-based analysis tool for the visualization and analysis of gene expression datasets. Boxplots were computed for individual cohorts, as well as combined cohorts. Significance of differential expression is computed using both *t*-tests and ANOVA with R statistical software (R version 3.6.1; http://www.r-project.org/).

### 2.14. Statistics

Results are expressed as mean ± SEM. *p*-value was determined by GraphPad Prism software 6 (GraphPad Software, La Jolla, CA, USA) using the unpaired student’s *t*-test. *p*  <  0.05 was considered significant.

## 3. Results

### 3.1. NEIL2 is Downregulated in the Human Colonic EDMs Following Fn Infection

**Figure 1:**
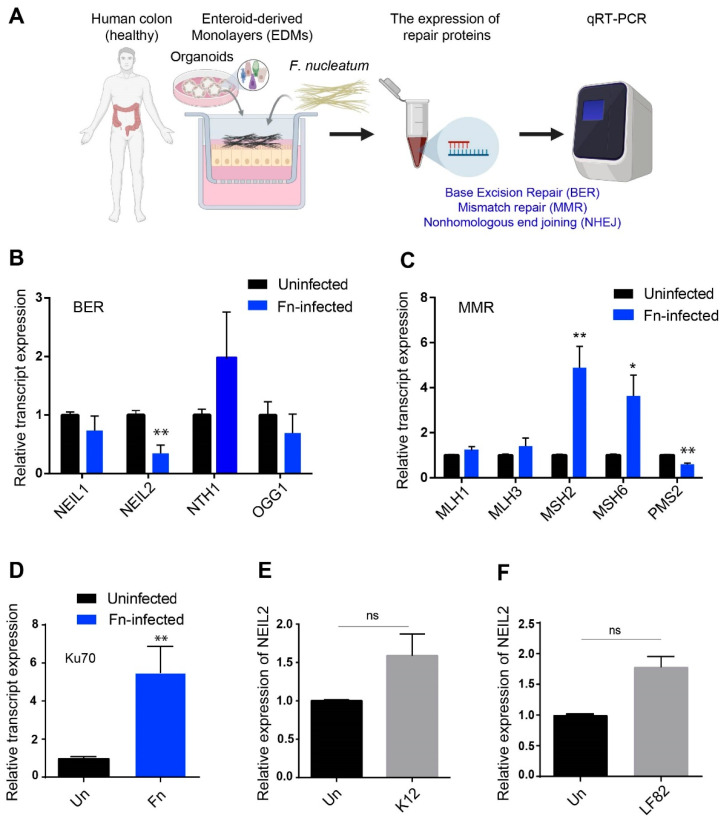
The specific downregulation of NEIL2 in human colonic EDMs following *Fusobacterium nucleatum* (*Fn*) infection. (**A**–**D**) Human colonic EDMs (from 3 healthy subjects) were infected with *Fn* at moi 100 for 24 h. (**A**) Schematic representation of the experimental design. The relative levels of BER transcripts NEIL1, NEIL2, NTH1, and OGG1 (**B**); MMR transcripts MLH1, MLH3, MSH2, MSH6, and PMS2 (**C**); and NHEJ marker Ku70 transcript (**D**) were determined by qRT-PCR. (**E**,**F**) Human colonic EDMs were infected with commensal *Escherichia coli*-K12 strain (**E**) or pathogenic IBD-associated adherent invasive *E. coli* LF-82 (**F**) to determine the expression level of NEIL2 following *Fn* infection. In (**B**–**F**), the expression level of the transcripts was normalized to the housekeeping gene (18srRNA), and the normalized expression value was compared with the respective uninfected control cells. Data represent the mean ± SEM of three separate experiments. * indicates *p* ≤ 0.05, and ** indicates *p* ≤ 0.01, as calculated by the unpaired two-tailed student’s *t*-test. ns: not significant.

To assess the expression levels of BER enzymes (Figure 1A), we infected colonic EDMs with *Fusobacterium nucleatum* (*Fn*) as a model pathogenic bacteria that are associated with CRC [18,19]. It was found that *Fn* infection significantly downregulated NEIL2 transcript level, while the NTH1 transcript level was upregulated, and the transcript levels of NEIL1 and OGG1 remained unaltered (Figure 1B). Since the mismatch repair (MMR) is involved in MSI CRCs, we thus examined the transcript levels of key components involved in MMR [53,54]. The levels of MLH1 and MLH3 remain unchanged after *Fn* infection, whereas the levels of MSH2 and MSH6 were increased, and PMS2 was downregulated following *Fn* infection (Figure 1C). Since *Fn* infection caused a decrease in the Ku70 level (a classical nonhomologous end joining (NHEJ) marker) in oral cancer cells [26], we tested its level as well in colon EDM and found that the upregulation of the Ku70 occurred following *Fn* infection (Figure 1D). These results thus demonstrate that *Fn* infection specifically suppressed the DNA glycosylase NEIL2, compared to all other repair proteins tested that are involved in various repair pathways. To demonstrate the specificity of the *Fn*-mediated downregulation of NEIL2, human colonic EDMs were infected with the commensal *E. coli*-K12 strain (Figure 1E) or IBD (Inflammatory bowel disease) -associated adherent invasive *E. coli* (*E. coli* LF82 isolated from IBD patients) (Figure 1F). The transcript level of NEIL2 was not affected following infection with these bacteria (Figure 1E,F), indicating the downregulation of NEIL2 following *Fn* infection is specific.

### 3.2. Fn Infection Suppressed the Expression of NEIL2 in the Murine Colonic EDMs

**Figure 2:**
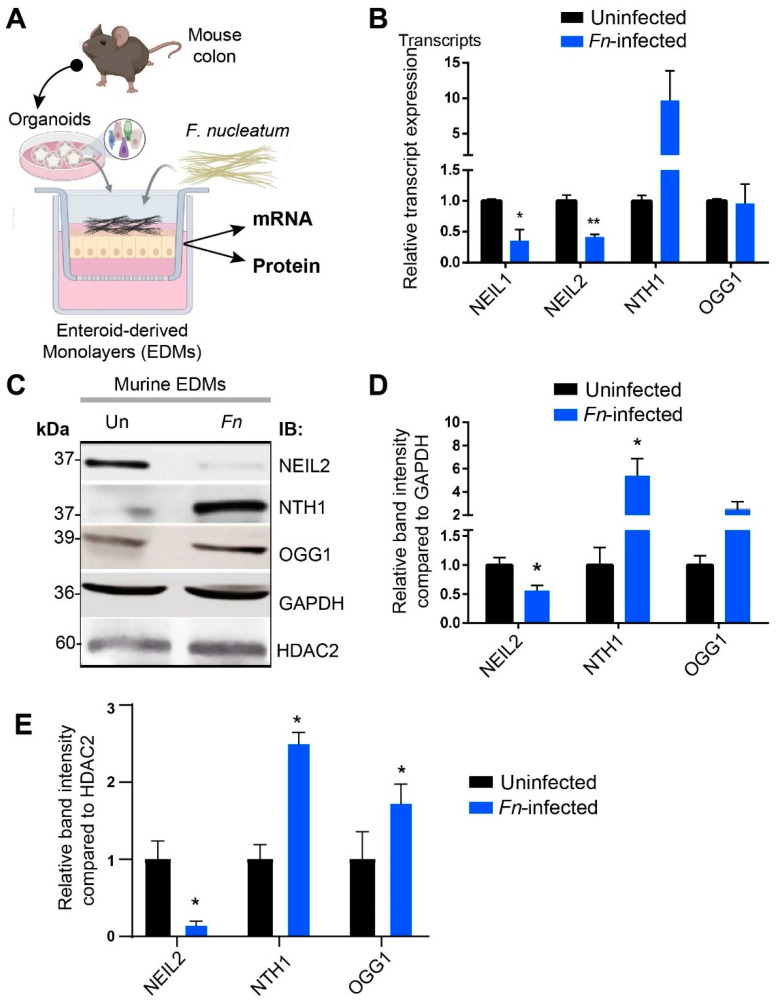
The downregulation of NEIL2 in murine colonic EDMs following *Fn* infection. (**A**) Schematic representation of the experimental design. (**B**) qRT-PCR to detect the expression levels of the BER enzymes following *Fn* infection of wild-type (WT) murine colonic EDMs for 24 h. The expression level of the transcripts was normalized to the 18srRNA and compared with the corresponding uninfected level. Data represent the mean ± SEM of three independent experiments. * and ** indicate *p* < 0.05 and *p* ≤ 0.01, respectively (**C**) Immunoblot to check the levels of NEIL2, NTH1, and OGG1 proteins in the whole-cell extract of uninfected and *Fn*-infected EDMs. GAPDH and HDAC2 (histone deacetylase 2) were used as loading controls. Three independent experiments (*n* = 3) were performed, and one representative figure is shown. Data represent the mean band density ± SEM. (**D**,**E**) The relative band intensity was normalized to both GAPDH (**D**) and HDAC2 (**E**) and compared between *Fn*-infected and uninfected, which was set as unity (*n* = 1). * indicate *p* < 0.05.

To determine the effect of *Fn* infection on the expression of BER proteins, we infected murine colonic EDMs with *Fn* and then measured the expression of DNA glycosylases at the transcript and protein levels (Figure 2A). Similar to human EDMs, *Fn* infection led to the downregulation of NEIL2 and NEIL1 transcripts. In contrast, OGG1 and NTH1 transcript levels were either unchanged or upregulated, respectively, probably as a compensatory host mechanism due to *Fn* infection (Figure 2B). Next, we assessed the expression level of BER proteins in the EDM cell lysates and found that *Fn* infection suppressed the expression of the NEIL2 protein. In contrast, the expression of NTH1 was upregulated significantly (Figure 2C–E).

### 3.3. The Role of NEIL2 in Controlling Inflammation and DNA Strand-Break Accumulation Following Fn Infection in Colonic EDMs 

**Figure 3:**
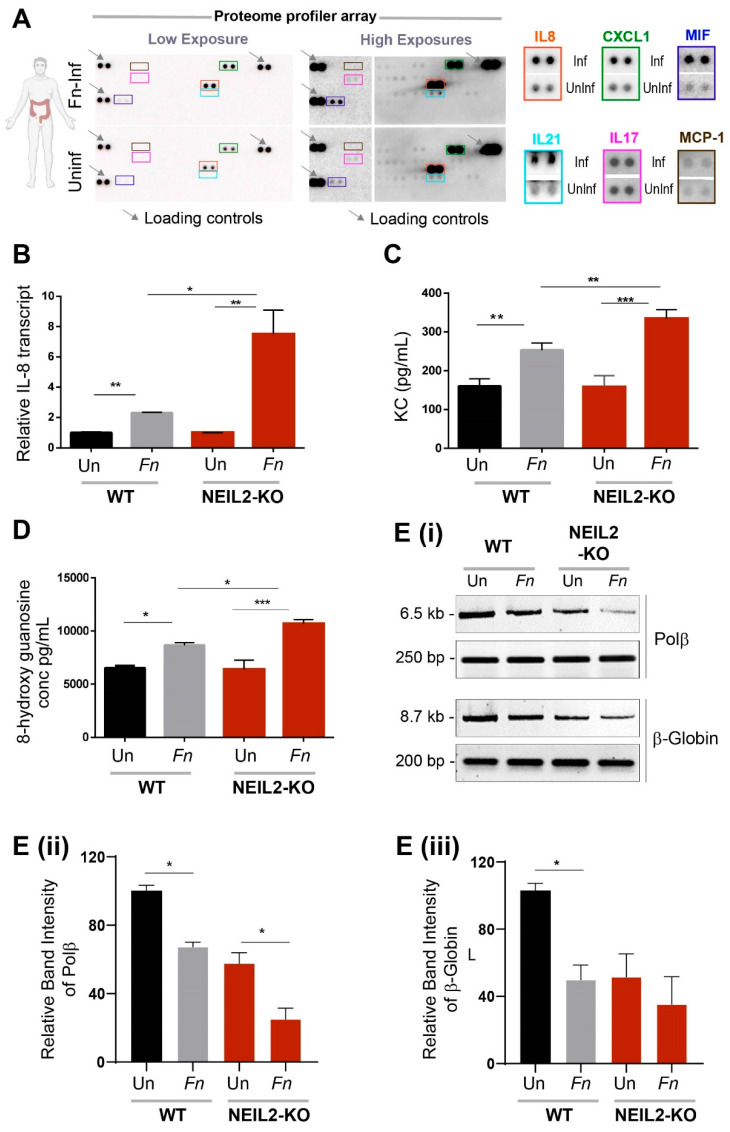
NEIL2 is crucial for limiting the inflammation and DNA damage induced by *Fn* infection. (**A**) Supernatants from uninfected and *Fn*-infected EDMs were analyzed for cytokines by proteome profiler arrays. Both low (left) and high (right) exposures were presented. Interleukin (IL)-8 and its homolog, CXCL1, were the major cytokines that were significantly upregulated in response to *Fn* infection, as shown in the boxes. (**B**) The differential expression of IL-8/Cxcl-1 was determined by qRT-PCR in WT and Neil2 knockout (KO) EDMs either untreated or infected with *Fn*. (**C**) The level of Cxcl-1/KC cytokine was measured in the supernatants from the EDMs of WT or *Neil2* KO mice either uninfected (Un) or infected with *Fn* by ELISA. (**D**) The supernatants of WT and Neil2 KO EDMs, either uninfected (Un) or infected with *Fn*, were analyzed for oxidative DNA/RNA damage. (**E**) (i) WT and Neil2 KO EDMs were either uninfected or infected with *Fn*, then harvested for genomic DNA isolation. Long amplicon quantitative-PCR (LA-qPCR) was performed to evaluate the level of DNA strand-break accumulation following *Fn* infection. Representative gels show the amplification of each long fragment (~7–8 kB) (upper panel) normalized to that of a short fragment (~250 bp) (lower panel) of the corresponding (Pol β and β-Globin) genes. The relative band intensity of Pol β (ii) and β-Globin (iii) was calculated. Data was generated from the mean ± SEM of three independent experiments, where * *p* < 0.05, ** *p* < 0.01, and *** *p* < 0.001.

To determine whether *Fn* infection can induce inflammatory cytokines, we collected supernatants from colonic EDMs, either mock-infected or infected with *Fn*. By analyzing the proteome profile array, we found that IL-8 and its homolog, CXCL1, were the major cytokines induced and secreted by *Fn-*infected EDMs (Figure 3A). The effect of NEIL2 on the expression of proinflammatory cytokines was further assessed using the WT vs. *Neil2* KO mouse EDMs following infection with *Fn.* It was found that *Fn* infection upregulated KC (IL-8) transcripts in both the EDMs; however, *NEIL2* KO EDMs had a four-fold higher KC compared to the WT EDMs following *Fn* infection (Figure 3B). Similarly, ELISA analyses also revealed that *Neil2* KO EDMs have a significantly higher KC expression compared to the WT EDMs following *Fn* infection (Figure 3C). As we have shown that NEIL2 prevents DNA damage accumulation following infection with *H. pylori* [44], we therefore examined the level of oxidized DNA bases in WT vs. *Neil2* KO EDMs. *Fn* infection indeed led to the accumulation of oxidized DNA base lesions, and *Fn*-infected *Neil2* KO EDMs accumulated a relatively higher level of oxidized DNA base lesions compared to *Fn* WT EDMs (Figure 3D). Furthermore, the level of DNA strand break was assessed using LA-qPCR in representative *Polβ* and *β-globin* genes in the genomic DNA extracted from colonic EDMs from WT and *Neil2* KO mice after the challenge with *Fn*. We indeed observed a significantly higher level of DNA strand-break accumulation in both the *Polβ and β-globin* genes in *Neil2* KO EDMs compared to WT EDMs following *Fn* infection (Figure 3E).

### 3.4. NEIL2 Knockdown Exacerbated Fn-Induced DNA Double-Strand Breaks (DSBs)

**Figure 4:**
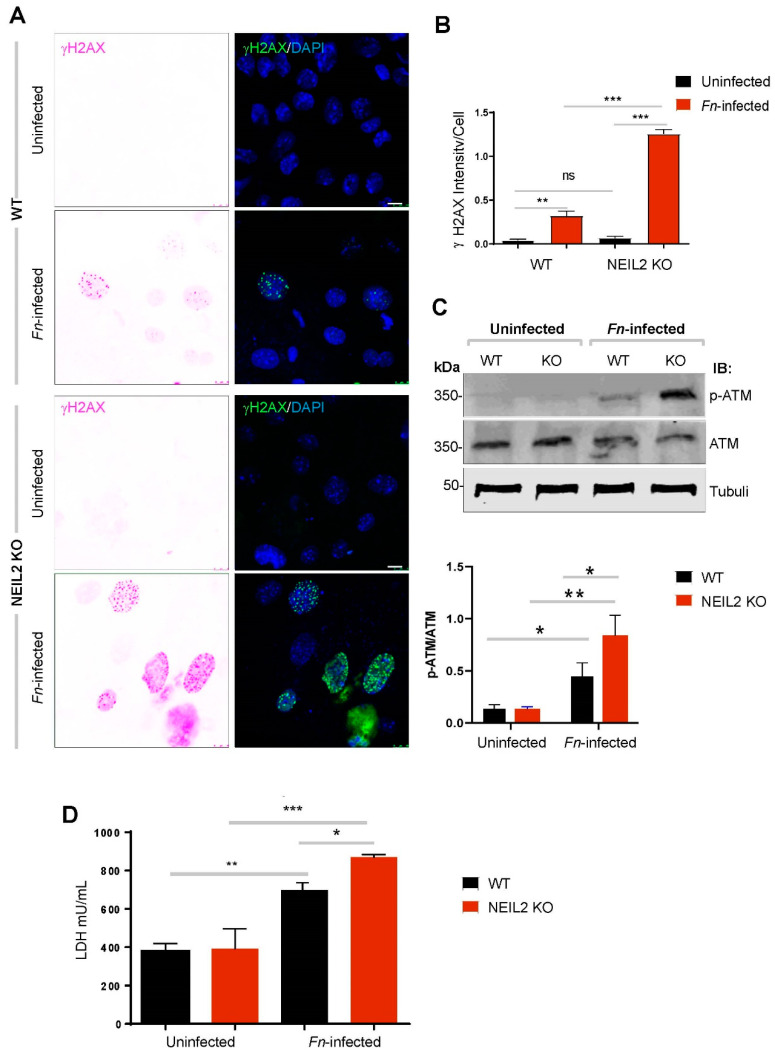
NEIL2 knockdown exacerbated *Fn*-induced DNA double-strand breaks (DSBs). (**A**) The level of γH2AX (a marker of DSBs, green) was determined by immunofluorescence (IF); DAPI was used for nuclei staining (blue). WT and *NEIL2* KO colonic EDMs either uninfected or infected with *Fn* were used to measure the amount of DSBs. (**B**) Using ImageJ, the intensity of γH2AX in each cell was calculated from all the four conditions shown in (**A**) and plotted. (**C**) The levels of phosphorylated Ataxia telangiectasia-mutated kinase (pATM) and total ATM was determined in whole cell lysates of both EDMs. Tubulin was used as a loading control (upper panel). The relative expression level of pATM normalized with those of total ATM were quantified by the ImageJ software. The expression level of pATM/ATM was compared in *Fn*-infected EDMs vs. uninfected EDMs and *Fn*-*NEIL2* KO EDMs vs. *Fn*-WT EDMs (lower panel). (**D**) LDH assay was performed on supernatants collected from WT and NEIL-2 KO EDMs either uninfected or infected with *Fn.* Data was generated from the mean ± SEM of three independent experiments. * indicates *p* < 0.05, ** *p* < 0.01, and *** *p* < 0.001.

Since *Fn* infection increased DNA strand breaks, we next investigated the formation of γH2AX foci, a marker of DSBs, by confocal microscopy and found that *Fn* infection induced the formation of γH2AX foci (Figure 4A). We then assessed the possible link between NEIL2 deficiency and DSB accumulation following *Fn* infection and found a significantly higher number of γH2AX foci in *Neil2* KO EDMs compared to WT EDMs (Figure 4A). The intensity of γH2AX/cell was calculated. As seen in Figure 4B, there is no difference in the intensity between WT and in *Neil2* KO EDMs in the baseline level (without infection). The intensity of γH2AX was increased after *Fn* infection, and a significantly higher intensity was recorded in *Neil2* KO EDMs compared to WT following *Fn* infection (Figure 4B).

Next, we assessed the effect of *Fn* infection on the activation of Ataxia telangiectasia-mutated kinase (ATM) protein by Western analysis. ATM is a serine/threonine kinase that regulates cell cycle checkpoints and DNA repair. ATM is activated by autophosphorylation (pATM) on Ser1981 in response to DSBs. We indeed found that *Fn* infection increased the expression of the pATM protein, and the ratio of pATM/total ATM was significantly higher in *Fn*-infected EDMs than the corresponding uninfected EDMs. Additionally, the expression of pATM was significantly higher in *Fn*-infected *NEIL2* KO EDMs compared to *Fn*-infected WT EDMs (Figure 4C), indicating DNA DSB accumulation in NEIL2-deficient cells.

To investigate the fate of *Fn*-infected EDMs and the degree of cell death, we measured the level of LDH in the culture media. We monitored the level of cell death using supernatants collected from WT and NEIL2 KO EDMs infected or not with *Fn*. Following *Fn* infection, cell death was increased, which was significantly higher in NEIL2 KO EDM compared to WT EDMs (Figure 4D).

### 3.5. Fn Infection Downregulated NEIL2 and Increased Oxidative Damage in the Murine CRC Model 

**Figure 5:**
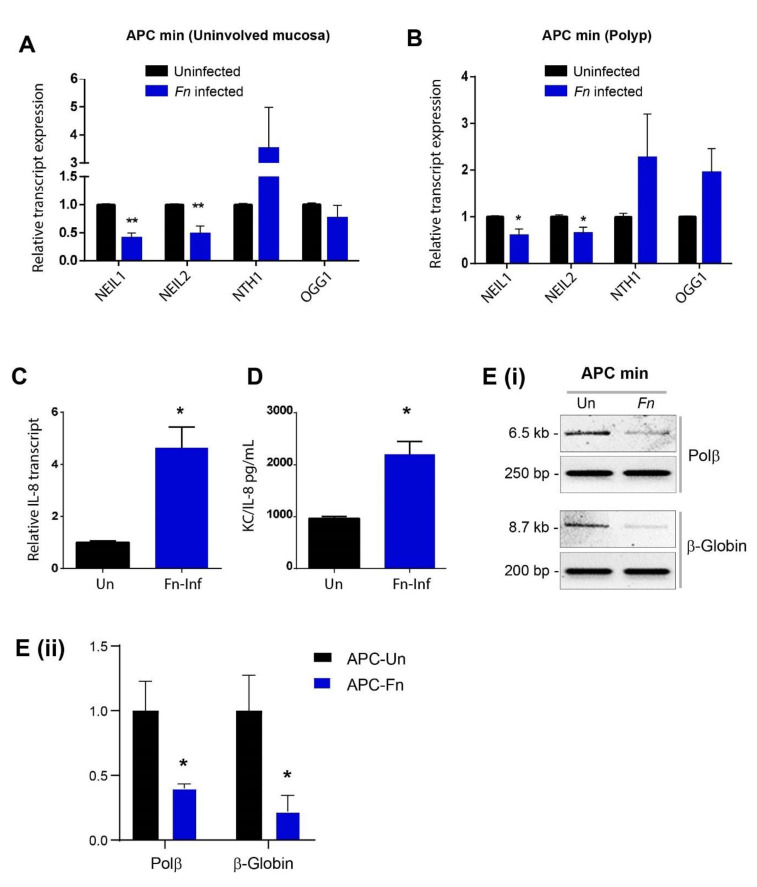
*Fn*-infection downregulated the NEIL2 transcript and increased the oxidative DNA damage in the murine colorectal cancer (CRC) model. (**A**) APC^Min/+^ EDMs derived from the uninvolved region of the colon were infected with *Fn* for 24 h, as previously described in the Methodology section; then, the level of BER transcripts (NEIL1, NEIL2, NTH1, and OGG1) was determined by qRT-PCR. Data represent the mean ± SEM of four separate experiments. ** indicates *p* ≤ 0.001. (**B**) APC^Min/+^ EDMs derived from the polyp region of the colon were infected with *Fn*; then, the level of BER transcripts (NEIL1, NEIL2, NTH1, and OGG1) was determined by qRT-PCR. Data represent the mean ± SEM of three separate experiments. * indicates *p* ≤ 0.05. (**C**) APC^Min/+^ EDMs derived from the polyp region of the colon were infected with *Fn*; then, the transcript and expression levels of KC/IL-8 were determined by qRT-PCR (**D**) The level of Cxcl-1/KC cytokine was measured in the supernatants from the EDMs of APC^Min/+^ EDMs derived from the polyp region either uninfected (Un) or infected with *Fn* by ELISA. (**E**) (i) LA-qPCR was performed to evaluate the level of DNA strand breaks after *Fn-*infection in APC^Min/+^ EDMs. The representative gels show the amplification of each long fragment (~7–8 kB) (upper panel) normalized to that of a short fragment (~250 bp) (lower panel) of the corresponding (*Pol β* and *β-Globin*) genes. (ii) The relative band intensity of *Pol β* and *β-Globin* was calculated. Data was generated from the mean ± SEM of three independent experiments where * *p* < 0.05.

To understand the effect of *Fn* infection on the expression of BER proteins in CRC, we analyzed their expression in CDX2 Cre APC^Min/+^ mice as the most commonly used murine CRC model. These mice developed multiple intestinal neoplasia and died within 8–12 weeks and were used to simulate human familial adenomatous polyposis (FAP) [55]. Additionally, *Fn* infection can increase the colon tumorgenesis in these mice in vivo [19]. The enteroids were isolated from the uninvolved region (normal, no polyp) and the involved/affected regions (polyps) of the colonic specimens of CDX2 Cre APC^Min/+^ mice. Similar to our previous observation in humans and WT murine EDMs, *Fn* infection suppressed the transcriptions of NEIL1 and NEIL2. In contrast, the transcriptions of NTH1 and OGG1 were either upregulated or unchanged in EDMs generated from uninvolved and involved regions (Figure 5A,B). *Fn* increased the inflammatory response in APC^Min/+^ polyp EDMs, as shown by the increase in KC/IL-8 expression by qRT-PCR and further confirmed by ELISA (Figure 5C,D). Next, we assessed the DNA strand-break levels in the *Polβ* and *β-globin* genes from APC^Min/+^ EDMs either untreated or infected with *Fn* using LA-qPCR (Figure 5E). We observed a significantly higher level of DNA strand-break accumulation in both the *Polβ and β-globin* genes following *Fn* infection compared to uninfected EDMs (Figure 5E).

**Figure 6:**
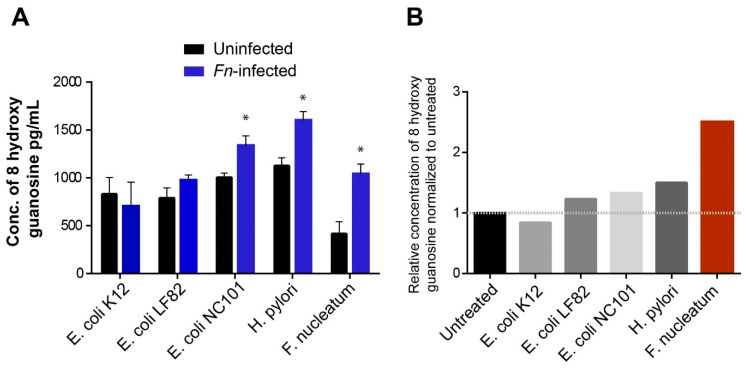
*Fn*-infection was the highest inducer of the oxidative DNA/RNA damage in the murine CRC model. (**A**) APC^Min/+^ EDMs derived from the uninvolved region of the colon were infected with different microbes: commensal E. coli K12; IBD-associated adherent-invasive *E. coli* LF82; and colon cancer-associated pathogens (NC101, *H. pylori*, and *Fn*). The infection with all the previous microbes was done for 24 h, and the supernatants were collected from the uninfected and infected EDMs from the same experiments and assessed for oxidative DNA damage (right). Data represent the mean ± SEM of three separate experiments. * indicates *p* ≤ 0.05. (**B**) The relative level of the oxidized bases generated by each microbe was normalized to the level of the oxidized bases in uninfected cells, which was arbitrarily set as 1, and the level of the oxidized bases in the infected EDMs was calculated. The relative production of the oxidized base was compared among different microbes, as indicated.

To determine the impact of *Fn* infection on oxidative damage generated in APC^Min/+^ EDMs, we measured the level of oxidatively damaged bases in the supernatants of APC^Min/+^ EDMs infected with *Fn* and compared with commensal *E. coli* K12 and other gut pathogens associated with CRC or IBD (Figure 6A). A significantly higher level of oxidative DNA base damages was produced by colon cancer-producing bacteria such as *E. coli* NC101 strain, *H. pylori*, and *Fn* (Figure 6A) than by commensal and other gut pathogens. When comparing the level of oxidized base adducts produced from different colon cancer pathogens to untreated EDMs, we found that *Fn* infection had the highest oxidative DNA damage levels, followed by *H. pylori* and *E. coli* NC101 (Figure 6B). Collectively, these results showed that *Fn* could suppress the expression of BER proteins NEIL2 in the murine CRC model, leading to an increase in the inflammatory response and an accumulation of DNA strand breaks, which could eventually contribute to the development of CRC.

### 3.6. NEIL2 Is Downregulated in the MSS-CRCs Patients 

**Figure 7:**
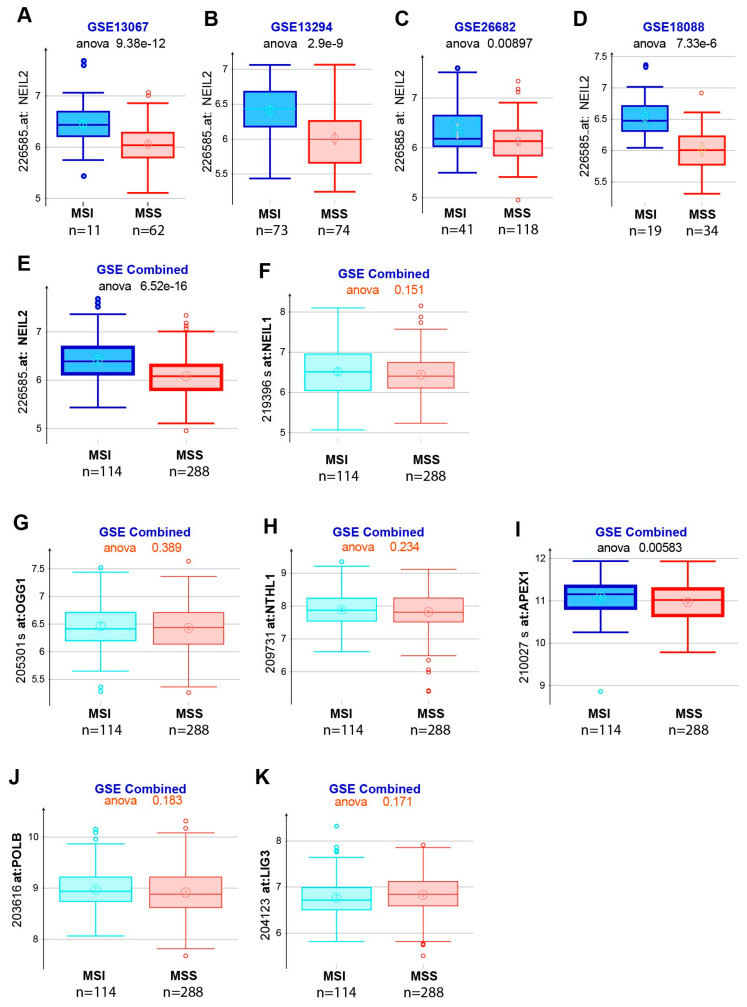
Analysis of NEIL2 in CRCs patients. The expression of BER proteins was analyzed in microsatellite instability (MSI) and microsatellite stable (MSS) groups from the different publicly available datasets GSE13067, GSE13294, GSE26682, and GSE18088 (**A**–**D**) The expression level of NEIL2 was determined in the above datasets. In the 4 combined datasets (GSE13067, GSE13294, GSE26682, and GSE18088), the expression levels of NEIL2 (**E**) NEIL1 (**F**), OGG1 (**G**), NTHL1 (**H**), APEX1 (**I**), Polb (**J**), and LigIII (**K**) were determined in between MSI and MSS CRCs.

CRC is classified into two categories: (i) the microsatellite instability-high (MSI-H) group, which has defects in the DNA mismatch repair (MMR) system and accounts for 15% of tumors, and (ii) the microsatellite stable (MSS) group, with proficient MMR proteins, which accounts for the remaining 85% of tumors [56,57,58]. Previously, a set of gastric cancer patients with and without microsatellite instability (named MSI and MSS, respectively) showed a differential expression profile of some of the DNA repair genes [59]. Here, we have examined the expression of several key BER proteins in MSI vs. MSS CRCs in four different publicly available datasets with GSE13067, GSE13294, GSE26682, and GSE18088 [60,61,62,63,64]. Interestingly, the analysis of the samples from the MSI vs. MSS group revealed that the level of NEIL2 (Figure 7A–E), but not other BER proteins (NEIL1, OGG1, NTHL1, APEX1, Polb, and LigIII; Figure 7F–K), is significantly decreased in the MSS group compared to the MSI group. Collectively, these results indicate the role of NEIL2 in MSS-CRCs, where mismatch repair proteins are efficient.

## 4. Discussion

Chronic infection and inflammation can cause DNA damage that can be linked to the development of cancer, including CRC. However, to date, there is no report on how colon cancer-associated bacteria such as *Fn* infection can impact DNA damage and inflammation and how the BER proteins play a critical role in the etiology of colorectal cancer. Using 3D stem cell-based organoid-derived monolayer models from humans and mice, we found that *Fn,* but not commensals or other gut pathogens, specifically suppressed the NEIL2-mediated BER pathway. Compared to WT EDMs, *NEIL2*-KO EDMs showed higher inflammatory cytokines (KC/IL-8) and a higher level of oxidative DNA/RNA damage following *Fn* infection. Notably, *Neil2*-KO EDMs showed higher levels of p-γH2AX and p-ATM after *Fn* infection, indicating the formation of DSBs. There is no evidence, to date, suggesting any direct role of NEIL2 in double-strand break repair (DSBR). We have reported earlier that NEIL2 is involved in transcription-coupled BER (TC-BER), where it remains associated with RNA polymerase II (RNAP II), transcription factors, and multiple repair proteins in a multi-protein complex [31,65]. We thus postulate that NEIL2-deficient cells following *Fn* infection accumulate DNA adducts, which can block DNA transcription and/or replication, leading to the generation of DSBs. 

Of note, NTH1, which has overlapping substrate specificities with NEIL2, was significantly upregulated following *Fn* infection, probably as a compensatory mechanism to mitigate NEIL2 deficiency. Such an overexpression of NTH1 can overwhelm the downstream repair process via an excessive incision of DNA lesions, resulting in the accumulation of BER intermediates. The accumulation of two unprocessed DNA strand breaks in close proximity in two opposing strands can also lead to DSB formation. Indeed, the previous report demonstrated that the overexpression of NTH1 could lead to DSB formation, inducing genome stability, and it does so by inhibiting homologous recombination-mediated DSBR [66]. Hence, a balance of the DNA glycosylases would be important to properly maintain genomic integrity. In the current study, we have focused on NEIL2 for the following reasons: (a) No significant difference was observed in embryonic cells with or without wild-type Nth1 following exposure to hydrogen peroxide [67]. In contrast, middle-to-old-aged *Neil2*-null mice showed the progressive accumulation of oxidative genomic damage, mostly in the transcribed regions, and furthermore, these mice were highly responsive to inflammatory agents [31]. (b) Among other BERs, only NEIL2 is significantly decreased in the MSS group compared to the MSI group (Figure 7). (c) Our current results showed that the measurement of 8-hydroxy guanosine (Figure 3D), LA-PCR (Figure 3E), and the accumulation of double-strand breaks by γH2AX staining (Figure 4A,B) conclusively showed a higher level of DNA strand-break accumulation in WT cells after *Fn* infection (with a lower NEIL2 expression level). Moreover, the level of DNA damage is significantly higher in *Neil2* KO mice infected EDMs compared to WT EDMs after *Fn* infection. 

Therefore, NEIL2 has an important role in reducing inflammation and DNA damage following infection. A recent study showed that *Fn* promoted oral squamous cell carcinoma (OSCC) by upregulating the expression of γH2AX and the accumulation of DSBs, leading to accelerating the cell cycle and increasing the cell proliferation capacity [26]. Similarly, *H. pylori* activated ATM due to the induced host genomic instabilities produced by cag PAI-positive strains through the accumulation of DSBs [68]. DSBs are considered to be the major source of chromosomal instability that can drive cancer development. Hence, exploring the mechanism of DSB formation, and the role of NEIL2 therein, is of great importance in understanding the etiology of CRC. Previous work has shown that the reduced expression of the NEIL2 protein is associated with the progression of several types of cancer, including CRC [37]. Additionally, lower NEIL2 expressions within each stage of the disease led to a lower probability of survival compared to patients with higher NEIL2 expressions [59]. Our results showed that *Fn* infection specifically suppressed NEIL2, which, in turn, increased DNA strand-break accumulation and inflammatory responses, leading to the initiation and progression of CRC. To the best of our knowledge, this is the first report where the DNA repair pathway and, specifically, the role of the DNA glycosylase NEIL2 has been extensively studied in the progression of CRC following *Fn* infection. Hence, this seminal observation of higher DSBs in the *Neil2* KO mice EDMs following infection with *Fn* might open up a new direction to study how NEIL2 deficiency can trigger DSB formation. 

APC^Min/+^ mice are an excellent experimental model for studying genetic, epigenetic, environmental, and therapeutic aspects of intestinal neoplasia in humans [69]. The oral feeding of *Fn* to APC^Min/+^ mice resulted in accelerated small intestinal and colonic tumorigenesis, the infiltration of specific myeloid-derived immune cell types into tumors, and an NF-κB proinflammatory gene signature similar to humans [19]. Other bacterial pathogens have been identified that promote colitis-associated CRC in APC^Min/+^ mice, such as *Bacteroides fragilis* and adherent-invasive *Escherichia coli* strain NC101 [70,71]. We thus studied the effect of *Fn* infection on BER in the colonic EDMs derived from APC^Min/+^ mice. We used both polyp and noninvolved (n.i.) regions of APC^Min/+^ where *Fn* infection downregulated NEIL2 and increased the inflammatory response and the extent of DNA strand-break accumulation. These observations were consistent with our results in human and murine subjects.

Previously, Tahara et al. reported the presence of *Fn* in cancer and in the adjacent normal regions [20]. The prevalence of high *Fn* was significantly elevated in CIMP (CpG island methylator phenotype)-positive CRCs compared to CIMP-negative cases. In addition, high *Fn* was significantly associated with molecular features that are common in CIMP CRCs, such as TP53 wild type, hMLH1 methylation positivity, and MSI [20]. This study showed that a large fraction *Fn* is also present in healthy normal and in MSI CRCs. NEIL2 was not reported in this study, and therefore, we did not correlate the level of NEIL2 in this specific study for *Fn* load and CRC subtypes. Further studies are required to understand the mechanism and any specific correlation between the level of NEIL2 following *Fn* association with MSI and MSS CRCs.

Furthermore, we investigated the expression of several other BER genes in MSI and MSS CRC cohorts using the publicly available data. Interestingly, only NEIL2 is downregulated in the MSS CRC cohort compared to the MSI cohort, consistent with our experimental observations. MSI occurs when the MMR genes stop functioning at their highest potential, and it is often associated with Lynch syndrome. The direct evidence regarding the link between BER and MMR was shown in MUTYH-associated polyposis (MAP), where MUTYH interacts with MMR gene products [72]. Genetic defects or inherited variations in BER proteins is a predisposing factor for the development of CRC [73,74]. Importantly, one novel missense variant of NEIL2 (C367A) was identified in 94 familial CRC cases but not in the 188 healthy control DNA [75]. The search for other variants, and detailed biochemical characterizations of those variants, are necessary to demonstrate the role of NEIL2 in CRC pathogenesis convincingly. DNA repair capacity via the BER pathway also represents a potential prognostic biomarker, and it is associated with patients’ survival and prediction of therapy responses [76]. More detailed mechanistic studies are required to understand the impact of *Fn* infection in CRC progression and the specific downregulation of the BER protein NEIL2 therein. Such studies will be helpful in effective treatment interventions of CRC and the outcome of therapy. 

## Figures and Tables

**Table 1:** Sequences of the primers used in the study. IL: interleukin.

Gene	Species	Forward Primer (5’ → 3’)	Reverse Primer (5′ → 3’)
18srRNA		GTAACCCGTTGAACCCCATT	CCATCCAATCGGTAGTAGCG
NEIL1	mouse	TCGTAGACATCCGTCGCTTT	TGTCTGATAGGTTCCGAAGTACG
NEIL2	mouse	CTGCCGCCTTTCAGTCTCT	TCTGGATCAAACCGAAGGAA
NTH1	mouse	GCATGAACTCAGGGAAGGAAGA	CCTCACCATTAGCCGCTTCA
OGG1	mouse	TTATCATGGCTTCCCAAACC	GTACCCCAGGCCCAACTT
NEIL1	human	CCAGGCAGTGGGAAGTCA	AGGGAGGGTGGCAGAGTC
NEIL2	human	GGGGCAGCAGTAAGAAGCTA	GGAATAATTTCTTTCCATGGACCT
NTH1	human	GACAGCATCCTGCAGACAGA	TTGATGTATTTCACCTTGCTCCT
OGG1	human	CCAGACCAACAAGGAACTGG	CAAATGCATTGCCAAGGA
IL-8/KC	mouse	CGCTTCTCTGTGCAGCGCTGCTGCT	AAGCCTCGCGACCATTCTTGAGTC
IL-8	Human	GAGCACTCCATAAGGCACAAA	ATGGTTCCTTCCGGTGGT
MLH1	Human	GATTACCCCTTCTGATTGACA	ACTGAGGCTTTCAAAACA
MLH3	Human	CGCACGAGCCTCAAGATCC	TCTGACTGGAAATAATTGCCTGGA
MSH2	Human	CAGTATATTGGAGAATCGCA	AGGGCATTTGTTTCACC
MSH6	Human	GCTTCTTCCCCAAGTCTCCG	AGAAGTCACAACTGGTGGGG
PMS2	Human	ACTGAGTCTAAGCACTGCGG	TGACATCGCTCAGTGCACAA
Ku70	Human	CCACAGGAAGAAGAGTTGGA	CTGCTCTGGAGTTGCCATGA

**Table 2:** The oligo sequences used in the study.

Primer Name	Gene Name	Sequences	PCR
m-*Polβ*-LA-F	*Polβ* (mouse)	TAT CTC TCT TCC TCT TCA CTT CTC CCC TGG	LA-qPCR
m-*Polβ*-LA-R	*Polβ* (mouse)	CGT GAT GCC GCC GTT GAG GGT CTC CTG	LA-qPCR
m-*β Globin*-LA-F	*β globin* (mouse)	TTG AGA CTG TGA TTG GCA ATG CCT	LA-qPCR
m-*β Globin*-LA-R	*β globin* (mouse)	CCT TTA ATG CCC ATC CCG GAC T	LA-qPCR
m-*Polβ*-SA-F	*Polβ* (mouse)	TATGGACCCCCATGAGGAACA	SA-PCR
m-*Polβ*-SA-R	*Polβ* (mouse)	AACCGTCGGCTAAAGACGTG	SA-PCR
m-*β Globin*-SA-F	*β globin* (mouse)	ACACTACTCAGAGTGAGACCCA	SA-PCR
m-*β Globin*-SA-R	*β Globin* (mouse)	ATACCCAATGCTGGCTCCTG	SA-PCR

## Abbreviations

EDTA	Ethylenediaminetetraacetic acid
TGFb	Transforming Growth Factor b
PVDF	Polyvinylidene difluoride
NFkB	Nuclear factor kappa-light-chain enhancer of activated B-cells
HEPES	4-(2-hydroxyethyl)-1-piperazineethanesulfonic acid
